# Exploring the relationship between secondhand smoke exposure in different indoor environments and depression symptoms among non-smoking adults: A cross-sectional study from NHANES

**DOI:** 10.18332/tid/207154

**Published:** 2025-08-28

**Authors:** Ying Liu, Jingtao Yu, Fanqiang Meng

**Affiliations:** 1 Beijing Key Laboratory of Mental Disorders, National Clinical Research Center for Mental Disorders, Beijing, China; 2 National Center for Mental Disorders, Beijing Anding Hospital, Capital Medical University, Beijing, China; 3 Peking University First Hospital, Beijing, China

**Keywords:** secondhand smoke, depression symptoms, cross-sectional study, NHANES

## Abstract

**INTRODUCTION:**

The existing evidence regarding the relationship between secondhand smoke (SHS) exposure and depression symptoms in non-smoking adults remains inconclusive. This cross-sectional study aims to further investigate this relationship using data from the National Health and Nutrition Examination Survey (NHANES).

**METHODS:**

SHS exposure was assessed through self-reported passive exposure to indoor tobacco products, such as those encountered at work or in vehicles. Depression symptoms were measured using the Patient Health Questionnaire-9 (PHQ-9) scale. Logistic regression and stratified analyses were conducted to evaluate the association between exposure to seven different indoor sources of SHS and depression symptoms.

**RESULTS:**

This study included 6272 never smoker adults from the US. Compared to individuals not exposed to any indoor SHS, exposure to specific types of SHS was positively associated with depression symptoms: exposure to cars (AOR=1.64; 95% CI: 1.17–2.31), exposure to other indoor areas (AOR=2.03; 95% CI: 1.33–3.10), and exposure to e-cigarettes (AOR=1.78; 95% CI: 1.14–2.77). When cumulative SHS exposure was calculated based on the number of SHS environments to which participants were exposed, those exposed to 1–2 sources of SHS were 1.47 times more likely (AOR=1.47; 95% CI: 1.13–1.91) and those exposed to ≥3 sources were 1.96 times more likely (AOR=1.96; 95% CI: 1.17–3.28) than unexposed individuals to experience depression symptoms.

**CONCLUSIONS:**

Exposure to specific SHS environments, particularly simultaneous exposure to multiple SHS environments, seems to be significantly associated with depression symptoms among US adults. Establishing causality and understanding the health implications of this connection will require future longitudinal investigations.

## INTRODUCTION

Depression is a prevalent mental disorder defined by a persistent low mood, diminished interest in activities, and decreased engagement in daily tasks^1^. In the United States, the prevalence of depression is approximately 5.9%, resulting in an annual economic burden exceeding $210 billion due to lost productivity and treatment expenses^1,2^.

Depression is a multifactorial disorder influenced by a variety of risk factors, including sociodemographic attributes, economic conditions, and behavioral factors. Recent research has drawn attention to modifiable risk factors, such as indoor air pollution from SHS, and their effects on mental health^3-5^. SHS, which refers to the inhalation of smoke from burning cigarettes or the exhaled smoke of smokers, is a hazardous mixture that contains 69 carcinogens and over 7000 harmful compounds^6^. It is responsible for approximately 41000 of the half a million tobacco-related deaths in the US each year^7^. The harmful substances in SHS may lead to chronic inflammation of the upper respiratory tract, thereby increasing the risk of chronic obstructive pulmonary disease, lung cancer, tuberculosis, and other respiratory conditions^8^. Epidemiological studies have demonstrated that exposure to SHS is independently associated with depression symptoms in women and children^9^, although findings regarding the general adult population remain conflicting.

A study conducted in Germany established a correlation between high exposure to SHS and current symptoms of depression in adults^10^. Conversely, research by Bot et al.^11^ involving two independent cohorts of non-smoking Dutch adults (totaling 2845 individuals) found no significant association between SHS exposure and depression symptoms. These conflicting results may stem from differences in the measurement methods used for SHS exposure and depression symptoms, as well as variations in research design. Consequently, additional epidemiological evidence regarding the effects of SHS on depression symptoms is warranted. Moreover, limitations such as reliance on outdated data and the focus on specific populations (e.g. perimenopausal women) may restrict the generalizability of findings to a broader demographic^12,13^. Notably, while exposure to SHS in family and workplace settings has garnered considerable attention, sources of exposure in diverse environments such as bars, restaurants, and vehicles have been less thoroughly examined^10-14^. This gap may impede the accurate identification of specific SHS exposure sources, potentially undermining public health policies aimed at effectively targeting high-risk areas. We hypothesize that exposure to SHS in various indoor environments may be positively associated with increased depression symptoms, even after adjusting for relevant covariates. Therefore, this study seeks to test this hypothesis using data from the National Health and Nutrition Examination Survey (NHANES) among the general adult population in the US.

## METHODS

### Study population

NHANES is a comprehensive and ongoing survey of the non-institutionalized population in the United States, employing stratified, multistage probability sampling techniques to gather nationally representative data on health and nutrition^15^. All NHANES protocols have received approval from the National Center for Health Statistics Research Ethics Review Board, and all participants have provided written informed consent. For additional information see (https://www.cdc.gov/nchs/nhanes/index.htm).

**Figure 1:**
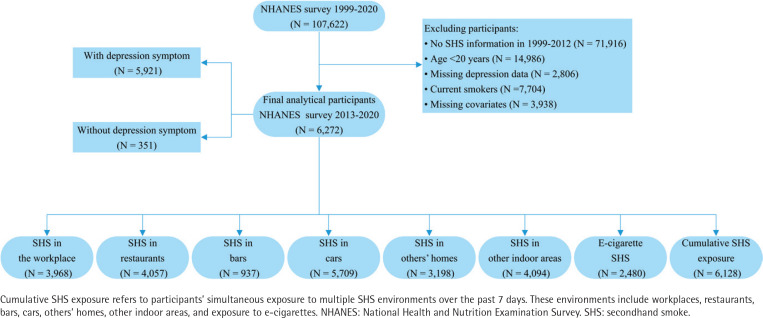
Flowchart of sample selection, NHANES 2013–2020 (N=6272)

**Figure 2:**
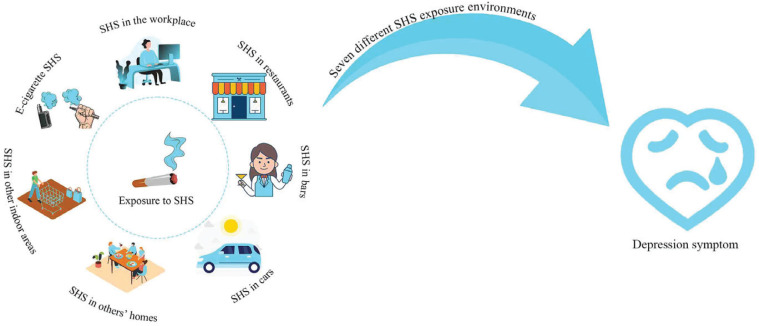
Conceptual framework: environmental pathways of secondhand smoke exposure and links to depression symptoms

Cross-sectional data from the NHANES collected between 2013 and 2020 were analyzed to ensure adequate sample size for robust statistical analyses and subgroup comparisons. From the complete NHANES dataset (1999–2020, n=107622), we excluded participants from 1999–2012 cycles lacking SHS information (n=71916) to focus exclusively on 2013–2020. We applied sequential exclusion criteria: individuals aged <20 years, totaling 14986 participants, were excluded to focus exclusively on an adult sample. Further exclusions included participants with missing depression data (n=2806), current smokers (n=7704), and those with incomplete covariate data (n=3938). Incomplete covariates included education level (n=7), marital status (n=4), poverty income ratio (PIR) (n=1130), BMI (n=96), sleep duration (n=29), physical activity level (n=1964), and alcohol use (n=708). Ultimately, this study comprised 6272 eligible participants, as illustrated in Figures 1 and 2.

### Assessment of exposure to SHS

SHS exposure among non-smokers was assessed using the NHANES 2013–2020 SHS Exposure Questionnaire. This questionnaire evaluates potential SHS exposure in various indoor environments, including workplaces, restaurants, bars, cars, others’ homes, and other indoor areas, over the past 7 days. Initial screening questions determined whether respondents had spent time in these environments. If they had, follow-up questions ascertained whether anyone else smoked or used tobacco products in those settings. If the answer was no, no further questions were posed. To evaluate exposure to vapors from e-cigarettes and other electronic nicotine delivery systems, respondents were asked: ‘In the past 7 days, have you ever been in an indoor environment where someone was using an e-cigarette, hookah, vape pen, or other similar electronic product?’^16^. Furthermore, we identified seven indoor environments with SHS exposure and categorized respondents according to the frequency of SHS encounters in the previous seven days: no SHS exposure, exposure to 1–2 instances of SHS, and exposure to three or more instances of SHS^17^.

### Assessment of depression symptoms

Depression symptoms were assessed using the Patient Health Questionnaire-9 (PHQ-9), a self-administered tool designed to screen for depression by evaluating symptoms experienced over the previous two weeks^18^. The PHQ-9 comprises nine items, each scored on a scale from 0 to 3, where 0 indicates no symptoms, 1 indicates symptoms present for a few days, 2 indicates symptoms occurring more than half the time, and 3 indicates symptoms experienced almost every day. The total PHQ-9 score ranges from 0 to 27, with higher scores reflecting more severe depression symptoms. Depression symptoms were dichotomized using a cut-off score of 10 or higher to identify the presence of clinically significant depression, as this threshold provides both sensitivity and specificity of 88% for major depression and represents the boundary between mild depression (scores 5–9) and moderate depression (scores 10–14)^19,20^.

### Covariates

In accordance with prior research^21^, potential confounders were adjusted by considering three key areas: sociodemographic characteristics, health status, and behavioral factors. Sociodemographic characteristics included age (20–39, 40–59, ≥60 years), gender (female, male), race/ethnicity (Mexican American, Non-Hispanic White, Non-Hispanic Black, and other race, including multi-racial and other Hispanic), education level (grade: <9, 9–11, and 12 without a diploma; High school graduate/GED or equivalent; Some college or Associate’s degree; and College graduate or higher), marital status (divorced/separated/widowed, single/never married, and married/living with a partner), and poverty income ratio [PIR: below poverty line (<1.0) and above poverty line (≥1.0)]^22^. Health status was assessed using BMI and sleep duration. BMI was calculated as weight (kg) divided by height squared (m^2^), with height and weight objectively measured by trained health technicians using standardized equipment in the Mobile Examination Center (MEC). BMI was categorized as <25, ≥25 to <30, and ≥30. Sleep duration was categorized as <7, ≥7 to <9, and ≥9. Behavioral factors included physical activity level (PA level), assessed using the Physical Activity Questionnaire (PAQ) based on the Global Physical Activity Questionnaire (GPAQ), and measured in metabolic equivalent (MET) minutes per week. MET values were calculated using standard coefficients of 8.0 METs for vigorous-intensity activities and 4.0 METs for moderate-intensity activities, with total weekly MET-minutes computed by summing across work-related, transportation, and recreational activity domains. Insufficient physical activity defined as <600 MET and sufficient physical activity as ≥600 MET^23^, and alcohol use (never, former, and current). Additional variables used in sensitivity analyses included hypertension (self-reported previous diagnosis by a doctor or health professional), diabetes mellitus (self-reported previous diagnosis by a doctor or health professional or current medication use), cardiovascular disease (self-reported physician diagnosis), sedentary behavior (self-reported sedentary time), total energy intake (assessed through 24-hour dietary recall), and serum cotinine levels (determined using isotope dilution high-performance liquid chromatography atmospheric pressure chemical ionization tandem mass spectrometry). Serum cotinine levels were categorized into tertiles (T1, T2, T3): T1 ≤0.011 ng/mL, T2 >0.011 to ≤ 0.037 ng/mL, and T3 >0.037 ng/mL.

### Statistical analysis

All statistical analyses were conducted using R version 4.4.1 (www.R-project.org). All tests were two-sided, with p<0.05 deemed statistically significant. The baseline characteristics of participants were summarized accordingly. Continuous variables are presented as mean ± standard error (SE), while categorical variables are reported as frequencies (n) and percentage (%). Group comparisons for continuous variables were performed using t-tests, and categorical variables were analyzed using chi-squared tests. To explore the association between exposure to seven different SHS environments and depression symptoms, multivariable logistic regression models were employed to calculate adjusted odds ratios (AOR) and 95% confidence intervals (CI). Three models were developed: an unadjusted model; Model 1, adjusted for age, gender, and race/ethnicity; and Model 2, further adjusted for BMI, poverty income ratio (PIR), marital status, education level, alcohol use, physical activity level, and sleep duration.

Stratified analyses were conducted based on sociodemographic characteristics, behavioral factors, and health status indicators. Interaction effects were tested to evaluate the consistency of these associations across subgroups. Sensitivity analyses were performed to validate the robustness of the models, incorporating additional adjustments for hypertension, diabetes mellitus, cardiovascular disease, sedentary behavior, total energy intake, and serum cotinine levels, as well as analyses with multiple imputation for missing covariates.

## RESULTS

### Basic characteristics of participants

Our study included a total of 6272 participants, with a mean age of 44.24 ± 0.39 years, of whom 46.1% (2787) were male. SHS exposure was absent in 78.4% of participants, while 18.8% and 2.7% were exposed to 1–2 and ≥3 environments, respectively. Among these participants, 351 (5.0%) reported experiencing depression symptoms. The prevalence of depression symptoms was significantly higher in the group exposed to ≥3 SHS environments compared to the no exposure group (p<0.001). As shown in Table 1, participants exhibiting depression symptoms were more likely to be female, have a lower PIR, and possess a higher BMI. With the exception of age, race, PA level, all other variables were significantly associated with depression symptoms (p<0.05). Supplementary file Figure S1 illustrates the linear relationships among the key covariates, with correlation coefficients ranging from -0.18 to 0.27, and most correlations being <0.20. Additionally, Supplementary file Tables S1 and S2 present the baseline characteristics stratified by gender and race/ethnicity, respectively.

**Table 1:** Characteristics of the study population stratified by depression symptoms, NHANES 2013–2020 (N=6272)

*Characteristics*	*Overall* *n (%)*	*Depression symptoms*		*p ^a^*
		*No* *n (%)*	*Yes* *n (%)*	
**Total participants**	6272 (100.0)	5921 (95.0)	351 (5.0)	
**Age** (years), mean ± SE	44.24 ± 0.39	44.35 ± 0.38	42.21 ± 1.25	0.069
20–39	2653 (43.8)	2500 (43.4)	153 (51.4)	0.082
40–59	2148 (35.8)	2039 (36.2)	109 (28.6)	
≥60	1471 (20.5)	1382 (20.5)	89 (20.1)	
**Gender**				**<0.05**
Male	2787 (46.1)	2669 (46.8)	118 (33.4)	
Female	3485 (53.9)	3252 (53.2)	233 (66.6)	
**Race/ethnicity**				0.269
Non-Hispanic White	2170 (64.2)	2054 (64.5)	116 (58.6)	
Non-Hispanic Black	1400 (11.0)	1320 (10.9)	80 (12.1)	
Mexican American	900 (9.1)	842 (9.0)	58 (9.8)	
Other race (including multi-racial, other Hispanic)	1802 (15.7)	1705 (15.5)	97 (19.5)	
**Education level**				**<0.001**
Less than grade 9	367 (2.8)	330 (2.6)	37 (5.9)	
9–11 grade (includes grade 12 with no diploma)	456 (4.5)	409 (4.4)	47 (7.3)	
High school graduate/GED or equivalent	1160 (18.6)	1087 (18.2)	73 (25.5)	
Some college or Associate’s degree	2027 (31.0)	1,905 (30.9)	122 (32.4)	
College graduate or higher	2262 (43.2)	2190 (44.0)	72 (28.9)	
**Marital status**				**<0.001**
Married/living with partner	3888 (66.3)	3723 (67.1)	165 (50.8)	
Never married	1406 (21.0)	1309 (20.6)	97 (28.0)	
Widowed/divorced/separated	978 (12.7)	889 (12.3)	89 (21.3)	
**PIR,** mean ± SE	3.34 ± 0.05	3.38 ± 0.05	2.50 ± 0.12	**<0.001**
<poverty line (<1.0)	1025 (10.6)	919 (9.9)	106 (24.3)	**<0.001**
≥poverty line (≥1.0)	5247 (89.4)	5002 (90.1)	245 (75.7)	
**BMI** (kg/m^2^), mean ± SE	28.99 ± 0.16	28.86 ± 0.16	31.52 ± 0.54	**<0.001**
<25	1876 (31.0)	1802 (31.3)	74 (24.7)	**<0.001**
≥25 to <30	1963 (31.8)	1870 (32.2)	93 (25.0)	
≥30	2433 (37.2)	2249 (36.5)	184 (52.3)	
**Sleep duration** (hours), mean ± SE	7.44 ± 0.03	7.45 ± 0.03	7.25 ± 0.13	**<0.001**
<7	1730 (23.2)	1601 (22.5)	129 (36.7)	**<0.001**
≥7 to <9	3554 (62.5)	3407 (63.6)	147 (42.3)	
≥9	988 (14.3)	913 (13.9)	75 (21.0)	
**PA level**				0.168
Insufficient PA (<600 MET)	1099 (16.0)	1022 (15.8)	77 (19.5)	
Sufficient PA (≥600 MET)	5173 (84.0)	4899 (84.2)	274 (80.5)	
**Alcohol use**				**<0.05**
Never	1181 (14.5)	1119 (14.7)	62 (11.3)	
Former	438 (5.4)	402 (5.2)	36 (9.2)	
Current	4653 (80.0)	4400 (80.1)	253 (79.5)	
**SHS in the workplace**				0.108
No	3563 (91.9)	3424 (92.1)	139 (86.9)	
Yes	405 (8.1)	383 (7.9)	22 (13.1)	
**SHS in restaurants**				0.694
No	3905 (97.0)	3727 (97.0)	178 (96.6)	
Yes	152 (3.0)	140 (3.0)	12 (3.4)	
**SHS in bars**				0.093
No	734 (84.3)	703 (84.8)	31 (73.4)	
Yes	203 (15.7)	185 (15.2)	18 (26.6)	
**SHS in cars**				**<0.001**
No	5216 (92.7)	4952 (93.2)	264 (83.4)	
Yes	493 (7.3)	440 (6.8)	53 (16.6)	
**SHS in others’ homes**				**<0.05**
No	2814 (90.7)	2675 (91.1)	139 (82.8)	
Yes	384 (9.3)	348 (8.9)	36 (17.2)	
**SHS in other indoor areas**				**<0.05**
No	3793 (94.3)	3615 (94.6)	178 (88.5)	
Yes	301 (5.7)	270 (5.4)	31 (11.5)	
**E-cigarette SHS indoors**				**<0.05**
No	2140 (86.3)	2021 (86.8)	119 (78.8)	
Yes	340 (13.7)	308 (13.2)	32 (21.2)	
**Cumulative SHS exposure^b^**				**<0.001**
Without SHS exposure	4658 (78.4)	4,435 (79.1)	223 (66.1)	
Exposure to 1–2 SHS environments	1274 (18.8)	1174 (18.3)	100 (28.1)	
Exposure to ≥3 SHS environments	196 (2.7)	176 (2.6)	20 (5.7)	

a P-values are calculated using chi-square and t-tests; bold values indicate statistical significance.

b Cumulative SHS exposure refers to participants’ simultaneous exposure to multiple SHS environments over the past 7 days. These environments include workplaces, restaurants, bars, cars, others’ homes, other indoor areas, and exposure to e-cigarettes. BMI: body mass index. GED: general equivalent diploma. MET: Metabolic equivalent. NHANES: National Health and Nutrition Examination Survey. PA: physical activity. PIR: poverty income ratio. SE: standard error. SHS: secondhand smoke.

### Association between exposure to SHS and depression symptoms

Table 2 illustrates the association between exposure to seven different SHS environments and depression symptoms. In the crude model, exposure to SHS in the workplace and restaurants was not significantly associated with depressive symptoms, while exposure in other settings showed significant associations. After adjusting for age, gender, and race (Model 1), workplace exposure became statistically significant, while restaurant exposure remained non-significant, and associations in other settings remained significant. In the fully adjusted model (Model 2), which further controlled for PIR, BMI, education level, marital status, alcohol use, sleep duration, and PA level, participants exposed to SHS in cars (AOR=1.64; 95% CI: 1.17–2.31), other indoor areas (AOR=2.03; 95% CI: 1.33–3.10), and to e-cigarettes (AOR=1.78; 95% CI: 1.14–2.77) continued to show significant associations with higher odds of depression symptoms. In contrast, exposure in workplaces (AOR=1.29; 95% CI: 0.79–2.12), restaurants (AOR=1.60; 95% CI: 0.85–3.01), bars (AOR=1.85; 95% CI: 0.96– 3.56) and in others’ homes (AOR=1.41, 95% CI: 0.93–2.15) did not demonstrate statistically significant associations. Furthermore, individuals exposed to 1–2 SHS environments (AOR=1.47; 95% CI: 1.13–1.91) and those exposed to ≥3 SHS environments (AOR=1.96; 95% CI: 1.17–3.28) exhibited an increased likelihood of depression symptoms by 47% and 96%, respectively.

**Table 2:** Association between exposure to SHS and depression symptoms in logistic regression models, NHANES 2013–2020 (N=6272)

*Exposure*	*Cases/Participants*	*Crude Model ^a^* *OR (95% CI)*	*p ^b^*	*Model 1^a^* *AOR (95% CI)*	*p ^b^*	*Model 2^a^* *AOR (95% CI)*	*p ^b^*
**SHS in the workplace**							
No ®	139/3563	1		1		1	
Yes	22/405	1.41 (0.89–2.25)	0.141	1.65 (1.02–2.65)	<0.05	1.29 (0.79–2.12)	0.214
**SHS in restaurants**							
No ®	178/3905	1		1		1	
Yes	12/152	1.79 (0.98–3.30)	0.060	1.75 (0.95–3.22)	0.074	1.60 (0.85–3.01)	0.142
**SHS in bars**							
No ®	31/734	1		1		1	
Yes	18/203	2.21 (1.21–4.03)	**<0.05**	1.98 (1.06–3.71)	**<0.05**	1.85 (0.96–3.56)	0.064
**SHS in cars**							
No ®	264/5216	1		1		1	
Yes	53/493	2.26 (1.66–3.08)	**<0.001**	2.39 (1.73–3.30)	**<0.001**	1.64 (1.17–2.31)	**<0.05**
**SHS in others’ homes**							
No ®	139/2814	1		1		1	
Yes	36/384	1.99 (1.36–2.92)	**<0.001**	2.19 (1.47–3.25)	**<0.001**	1.41 (0.93–2.15)	0.104
**SHS in other indoor areas**							
No ®	178/3793	1		1		1	
Yes	31/301	2.33 (1.56–3.48)	**<0.001**	2.59 (1.71–3.90)	**<0.001**	2.03 (1.33–3.10)	**<0.05**
**E-cigarette SHS indoors**							
No ®	119/2104	1		1		1	
Yes	32/340	1.76 (1.17–2.65)	**<0.05**	1.77 (1.15–2.70)	**<0.05**	1.78 (1.14–2.77)	**<0.05**
**Cumulative SHS exposure^c^**							
Without SHS exposure ®	223/4658	1		1		1	
Exposure to 1–2 SHS environments	100/1274	1.69 (1.33–2.16)	**<0.001**	1.83 (1.42–2.35)	**<0.001**	1.47 (1.13–1.91)	**<0.05**
Exposure to ≥3 SHS environments	20/196	2.26 (1.40–3.66)	**<0.001**	2.61 (1.59–4.28)	**<0.001**	1.96 (1.17–3.28)	**<0.05**

a Crude Model: unadjusted; AOR: adjusted odds ratio. Model 1: adjusted for age, gender and race/ethnicity. Model 2: adjusted as for Model 1plus PIR, BMI, education level, marital status, alcohol use, sleep duration, and PA level.

b P-values in bold statistically significant.

c Cumulative SHS exposure refers to participants’ simultaneous exposure to multiple SHS environments over the past 7 days. These environments include workplaces, restaurants, bars, cars, others’ homes, other indoor areas, and exposure to e-cigarettes. BMI: body mass index. NHANES: National Health and Nutrition Examination Survey. PA: physical activity. PIR: poverty income ratio. SHS: secondhand smoke. ® Reference categories.

### Subgroup analysis and sensitivity analyses

The results of the independent subgroup analyses of various demographic variables are presented in Table 3. We observed that the association between exposure to SHS and depression symptoms was stronger in women than in men, with women showing significant associations for SHS exposure in cars (AOR=1.68; 95% CI: 1.10–2.57), e-cigarette SHS indoors (AOR=1.98; 95% CI: 1.12–3.52), and cumulative SHS exposure (AOR=1.51; 95% CI: 1.09–2.10), while men showed significant associations only for SHS in other indoor areas (AOR=2.65; 95% CI: 1.41–4.98). These analyses also revealed that race/ethnicity and education level modified the association between SHS exposure and depression symptoms (p for interaction <0.05). The association between cumulative SHS exposure and depression symptoms remained consistent across all stratified analyses, with no significant interactions observed (all p>0.05).

**Table 3:** Subgroup analyses of the association between exposure to SHS and depression symptoms, NHANES 2013–2020 (N=6272)

*Subgroup variables*	*Participants n (%)*	*SHS in the workplace*		*SHS in restaurants*		*SHS in bars*		*SHS in cars Exposure to 1–2 SHS*		*SHS in others’ homes Exposure to ≥3 SHS*		*SHS in other indoor areas*		*E-cigarette SHS indoors*		*Cumulative SHS exposure ^a^*		
		*AOR (95% CI) ^b^*	*p for interaction* *^c^*	*AOR (95% CI)^b^*	*p for interaction* *^c^*	*AOR (95% CI)^b^*	*p for interaction* *^c^*	*AOR (95% CI)^b^*	*p for interaction* *^c^*	*AOR (95% CI)^b^*	*p for interaction* *^c^*	*AOR (95% CI)^b^*	*p for interaction* *^c^*	*AOR (95% CI)^b^*	*p for interaction* *^c^*	*AOR (95% CI)^b^*	*AOR (95% CI)^b^*	*p for interaction* *^c^*
**Age** (years)			**0.026**		0.617		0.841		0.704		0.695		0.412		0.814			0.068
20–39	2653 (43.8)	1.46 (0.75–2.85)		2.25 (0.92–5.52)		1.99 (0.90–4.41)		1.72 (1.11–2.66)*		1.79 (1.03–3.09)*		2.22 (1.24–3.99)*		1.79 (1.01–3.17)*		2.19 (1.51–3.19)**	2.64 (1.39–5.01)*	
40–59	2148 (35.8)	0.61 (0.22–1.70)		1.64 (0.54–4.97)		1.75 (0.40–7.73)		1.43 (0.71–2.85)		0.84 (0.33–2.12)		1.26 (0.49–3.24)		1.32 (0.53–3.29)		0.84 (0.49–1.43)	1.15 (0.38–3.47)	
≥60	1471 (20.5)	4.89 (1.29–18.59)*		0.89 (0.18–4.47)		-		2.00 (0.78–5.14)		1.96 (0.72–5.35)		2.39 (0.94–6.09)		2.01 (0.51–7.93)		1.29 (0.72–2.31)	3.45 (0.62–19.35)	
**Gender**			0.686		0.734		0.638		0.669		0.858		0.262		0.377			0.493
Male	2787 (46.1)	1.04 (0.51–2.11)		1.26 (0.42–3.77)		1.50 (0.54–4.19)		1.56 (0.88–2.76)		1.29 (0.64–2.59)		2.65 (1.41–4.98)*		1.39 (0.68–2.85)		1.33 (0.86–2.06)	1.37 (0.58–3.24)	
Female	3485 (53.9)	1.54 (0.77–3.08)		1.73 (0.79–3.77)		2.29 (0.93–5.65)		1.68 (1.10–2.57)*		1.45 (0.85–2.44)		1.59 (0.88–2.87)		1.98 (1.12–3.52)*		1.51 (1.09–2.10)*	2.33 (1.21–4.50)*	
**Race/ethnicity**			0.677		0.424		0.126		0.135		**0.033**		0.824		0.164			0.397
Non-Hispanic White	2170 (64.2)	2.22 (0.91–5.41)		0.41 (0.05–3.28)		0.48 (0.08–2.69)		1.47 (0.82–2.65)		2.43 (1.25–4.72)*		1.72 (0.79–3.72)		3.44 (1.63–7.25)*		1.46 (0.92–2.32)	3.08 (1.24–7.69)*	
Non-Hispanic Black	1400 (11.0)	1.18 (0.41–3.38)		1.49 (0.42–5.33)		1.84 (0.50–6.76)		1.06 (0.56–2.01)		0.76 (0.32–1.81)		2.85 (1.30–6.24)*		1.82 (0.78–4.25)		1.14 (0.67–1.95)	1.54 (0.64–3.74)	
Mexican American	900 (9.1)	1.02 (0.33–3.18)		1.84 (0.47–7.26)		7.56 (0.42–135.75)		2.23 (0.83–5.98)		0.54 (0.12–2.49)		1.66 (0.48–5.77)		0.89 (0.20–3.99)		1.12 (0.56–2.22)	1.29 (0.26–6.41)	
Other race (including multi-racial, other Hispanic)	1802 (15.7)	1.06 (0.38–2.97)		2.68 (0.93–7.68)		4.90 (1.08–22.17)*		2.53 (1.23–5.21)*		1.55 (0.63–3.79)		1.47 (0.56–3.87)		0.74 (0.26–2.13)		1.89 (1.13–3.17)*	1.62 (0.52–5.05)	
**Education level**			0.205		0.942		0.470		**0.005**		0.889		0.580		**0.029**			0.236
Less than grade 9	367 (2.8)	0.48 (0.08–2.81)		1.54 (0.09–25.16)		-		2.66 (0.65–10.83)		0.23 (0.01–6.47)		0.88 (0.06–13.37)		-		0.87 (0.31–2.40)	1.26 (0.11–15.21)	
9–11 grade (Includes grade 12 with no diploma)	456 (4.5)	0.72 (0.19–2.78)		0.87 (0.09–8.16)		1.73 (0.07–40.35)		1.85 (0.71–4.82)		1.13 (0.38–3.39)		2.67 (0.72–9.83)		-		0.85 (0.39–1.82)	1.33 (0.35–5.08)	
High school graduate/GED or equivalent	1160 (18.6)	0.60 (0.17–2.14)		2.04 (0.53–7.84)		2.70 (0.23–31.61)		0.55 (0.24–1.30)		1.49 (0.57–3.89)		1.19 (0.43–3.27)		3.12 (1.38–7.06)**		0.94 (0.53–1.68)	1.20 (0.40–3.66)	
Some College or Associate’s degree	2027 (31.0)	1.88 (0.83–4.23)		2.02 (0.68–6.06)		1.20 (0.38–3.79)		1.95 (1.15–3.31)*		1.46 (0.77–2.76)		2.17 (1.11–4.20)*		1.27 (0.60–2.68)		1.77 (1.15–2.70)*	2.02 (0.90–4.54)	
College graduate or higher	2262 (43.2)	4.33 (1.51–12.42)*		1.51 (0.43–5.25)		3.45 (0.99–12.04)		4.42 (1.96–9.97)**		1.98 (0.71–5.53)		3.06 (1.15–8.10)*		2.26 (0.93–5.51)		2.60 (1.49–4.55)**	3.99 (1.27–12.54)*	
**Marital status**			0.288		0.597		0.985		0.406		0.401		**0.028**		0.660			0.861
Married/living with partner	3888 (66.3)	0.68 (0.28–1.67)		1.06 (0.31–3.58)		2.18 (0.69–6.82)		1.80 (1.05–3.09)*		1.76 (0.90–3.44)		0.82 (0.34–1.97)		1.74 (0.88–3.43)		1.29 (0.87–1.91)	1.14 (0.39–3.29)	
Never married	1406 (21.0)	1.88 (0.85–4.17)		1.85 (0.57–5.98)		2.05 (0.72–5.88)		1.18 (0.67–2.10)		1.01 (0.49–2.05)		3.52 (1.72–7.18) **		2.42 (1.17–5.01)*		1.68 (1.04–2.71)*	2.72 (1.31–5.63)*	
Widowed/divorced/separated	978 (12.7)	1.86 (0.65–5.33)		1.94 (0.64–5.85)		1.87 (0.30–11.49)		1.64 (0.79–3.43)		1.30 (0.55–3.08)		2.45 (1.04–5.75)*		0.76 (0.22–2.66)		1.31 (0.75–2.28)	1.38 (0.43–4.38)	
**PIR**			0.715		0.177		0.602		0.117		0.687		0.779		0.424			0.610
<poverty line (<1.0)	1025 (10.6)	1.08 (0.43–2.74)		3.30 (0.91–11.92)		1.95 (0.19–20.26)		2.48 (1.41–4.35)*		1.63 (0.77–3.45)		2.33 (1.07–5.10)*		1.33 (0.53–3.34)		1.40 (0.85–2.31)	2.72 (1.19–6.24)*	
≥poverty line (≥1.0)	5247 (89.4)	1.44 (0.80–2.59)		1.31 (0.61–2.78)		1.67 (0.81–3.47)		1.35 (0.86–2.10)		1.39 (0.83–2.34)		1.91 (1.15–3.19)*		1.93 (1.16–3.22)*		1.49 (1.10–2.03)*	1.54 (0.79–3.00)	
**BMI** (kg/m^2^)			0.305		0.919		0.775		0.181		0.431		0.433		0.550			0.262
<25	1876 (31.0)	1.77 (0.71–4.41)		1.45 (0.31–6.76)		2.52 (0.70–9.07)		2.21 (1.12–4.35)*		1.84 (0.78–4.35)		2.67 (1.16–6.13)*		**2.94 (1.15–7.55)**		2.36 (1.35–4.13)*	2.47 (0.97–6.26)	
≥25 to < 30	1963 (31.8)	1.56 (0.63–3.87)		1.44 (0.41–5.09)		1.33 (0.37–4.81)		1.54 (0.75–3.18)		1.08 (0.40–2.90)		2.52 (1.10–5.78)*		1.32 (0.52–3.35)		1.36 (0.82–2.26)	1.68 (0.59–4.72)	
≥30	2433 (37.2)	1.01 (0.45–2.27)		1.89 (0.81–4.42)		2.77 (0.90–8.54)		1.46 (0.90–2.36)		1.44 (0.83–2.52)		1.49 (0.77–2.88)		1.81 (0.97–3.38)		1.26 (0.87–1.81)	1.72 (0.78–3.76)	
**Sleep duration** (hours)			0.096		0.925		0.511		0.470		0.717		0.927		0.962			0.243
<7	1730 (23.2)	2.62 (1.30–5.29)*		1.70 (0.62–4.67)		**2.99 (1.12–8.00)**		1.45 (0.81–2.60)		1.18 (0.62–2.26)		2.57 (1.32–5.02)*		1.62 (0.73–3.61)		1.15 (0.74–1.81)	2.57 (1.26–5.26)*	
≥7 to < 9	3554 (62.5)	0.84 (0.34–2.06)		1.63 (0.61–4.37)		1.28 (0.44–3.76)		2.28 (1.35–3.85)*		1.84 (0.94–3.57)		1.69 (0.79–3.62)		1.56 (0.79–3.09)		1.62 (1.09–2.43)*	1.96 (0.84–4.60)	
≥9	988 (14.3)	0.70 (0.15–3.32)		1.95 (0.36–10.56)		-		1.17 (0.55–2.51)		1.44 (0.48–4.29)		1.71 (0.65–4.48)		1.64 (0.63–4.27)		1.68 (0.97–2.91)	0.47 (0.06–3.66)	
**PA level**			0.119		**0.009**		0.626		**0.015**		0.471		0.479		0.052			0.773
Insufficient PA (<600 MET)	1099 (16.0)	-		-		-		0.59 (0.19–1.79)		1.82 (0.64–5.16)		3.44 (1.29–9.14)*		3.92 (1.43–10.75)*		1.33 (0.71–2.48)	2.81 (0.72–10.98)	
Sufficient PA (≥600 MET)	5173 (84.0)	1.36 (0.83–2.24)		2.05 (1.08–3.88)		1.64 (0.80–3.33)		1.87 (1.30–2.68)**		1.33 (0.84–2.12)		1.88 (1.16–3.04)*		1.51 (0.91–2.50)		1.46 (1.10–1.95)*	1.72 (0.99–2.98)	
**Alcohol use**			0.422		0.229		0.932		**0.004**		0.568		0.190		0.774			0.203
Never	1181 (14.5)	2.20 (0.53–9.13)		7.08 (1.54–32.54)*		-		3.05 (1.18–7.91)*		1.97 (0.67–5.81)		4.60 (1.57–13.46)*		2.72 (0.22–33.38)		1.64 (0.83–3.25)	8.79 (1.71–45.25)*	
Former	438 (5.4)	-		1.82 (0.18–18.69)		-		**-**		0.81 (0.16–4.19)		5.49 (0.74–40.67)		-		0.77 (0.26–2.27)	-	
Current	4653 (80.0)	1.21 (0.71–2.08)		1.29 (0.60–2.76)		1.77 (0.91–3.42)		1.69 (1.17–2.45)**		1.35 (0.83–2.18)		1.68 (1.02–2.77)*		1.78 (1.13–2.81)*		1.46 (1.09–1.96)*	1.72 (0.99–2.99)	
**Serum cotinine** (ng/mL)			0.206		0.157		0.427		0.069		0.160		0.953		0.567			0.455
T1 ≤0.011	2567 (42.9)	0.29 (0.04–2.24)		0.42 (0.06–3.18)		3.98 (0.85–18.62)		2.34 (0.78–7.04)		0.25 (0.03–1.94)		2.03 (0.77–5.39)		1.49 (0.48–4.60)		1.19 (0.69–2.08)	-	
T2 >0.011 to ≤0.037	1454 (24.3)	1.23 (0.38–3.98)		1.16 (0.25–5.50)		1.47 (0.04–50.55)		3.98 (1.68–9.44)*		1.58 (0.53–4.68)		1.95 (0.62–6.12)		0.67 (0.18–2.49)		1.28 (0.70–2.35)	2.30 (0.48–10.91)	
T3 >0.037	1968 (32.9)	1.52 (0.81–2.87)		2.46 (1.07–5.64)*		2.12 (0.90–5.02)		1.29 (0.84–1.99)		1.57 (0.91–2.70)		2.09 (1.19–3.66)*		1.77 (0.97–3.25)		1.51 (1.02–2.23)*	2.30 (1.23–4.30)*	

a Cumulative SHS exposure refers to participants’ simultaneous exposure to multiple SHS environments over the past 7 days. These environments include workplaces, restaurants, bars, cars, others’ homes, other indoor areas, and exposure to e-cigarettes.

b Multivariable-adjusted model; AOR: adjusted odds ratio; each stratification controlled for all factors (age, gender, race/ethnicity, education level, PIR, marital status, alcohol use, sleep duration, BMI, PA level) except the stratification factor itself.

c The interaction test assesses the relationship between SHS exposure and participant characteristics. GED: General equivalent diploma. MET: metabolic equivalent. NHANES: National Health and Nutrition Examination Survey. PA: physical activity. PIR: poverty income ratio. SHS: secondhand smoke. T: tertiles.

* p<0.05;

** p<0.001.

The sensitivity analysis yielded results that were consistent with the primary findings (Supplementary file Table S5). Following additional adjustments for diabetes mellitus, cardiovascular disease, hypertension, sedentary behavior, and total energy intake across all participants, the association between exposure to SHS and depression symptoms remained statistically significant. Additionally, the multiple imputation of missing covariates did not alter the findings.

## DISCUSSION

This present nationally representative population-based investigation revealed that individuals exposed to SHS were more likely to experience symptoms of depression compared to those who were not exposed. This potential association was particularly pronounced in environments such as cars and other indoor settings, as well as in cases of simultaneous exposure to multiple sources of SHS. These findings underscore SHS exposure as a significant modifiable risk factor for depression symptoms.

Our findings align with previous epidemiological studies indicating that non-smokers exposed to SHS are positively associated with an increased likelihood of developing depression symptoms. A cross-sectional analysis from the Behavioral Risk Factor Surveillance System (BRFSS) reveals that non-smoking adults aged ≥18 years who are exposed to SHS in their homes and workplaces face an elevated risk of severe depression^24^. Similarly, recent data from a retrospective cohort study involving over 4000 participants aged ≥60 years from the Chinese Longitudinal Healthy Longevity Survey (CHARLS) suggest that both early and cumulative lifetime exposure to SHS is linked to a heightened risk of depression symptoms in the elderly^25^. Most earlier studies have concentrated on the relationship between exposure to SHS in home and occupational settings and the development of depression symptoms. Our research distinguishes among various exposure environments, thereby providing more detailed data. Specifically, we found a significant association between SHS exposure in cars and depression symptoms. This may be attributed to the confined space of vehicles, which leads to higher concentration of tobacco toxins and unavoidable exposure. Continente et al.^26^ demonstrated that nicotine concentrations in vehicles where smoking occurs reach extremely high levels (median 21.44 μg/m^3^), far exceeding those found in homes with smokers and even in hospitality venues before smoke-free policies^26^. In contrast, no association was identified between SHS exposure in restaurants and depression symptoms, which may be due to the lower frequency and shorter duration of exposure in these settings^27^. Participants with cumulative SHS exposure were significantly more susceptible to symptoms of depression compared to those with no SHS exposure or with fewer types of SHS exposure. These findings suggest a dose-response relationship, indicating that both the concentration and frequency of SHS exposure are linked to an increased risk of depression symptoms, potentially indicating a safety threshold at certain exposure levels^13^. The observed correlation between SHS exposure and an elevated odds ratio for depression symptoms may be attributed to several potential mechanisms. Firstly, it can be posited that nicotine exposure may have long-term effects on the dopamine system, resulting in persistent imbalances in dopamine transport^28^. This could subsequently heighten the risk of negative emotional states or depression. Furthermore, nicotine intake from SHS may influence dopamine levels in the brain. The dysregulation of the dopamine system due to nicotine addiction may increase the vulnerability of exposed individuals to depression^29^. Secondly, SHS exposure may reflect stress in living and working environments, which could exacerbate depression symptoms by impairing neuroplasticity mechanisms^18^. Chronic inflammation and neurobiological mechanisms further elucidate this association. Finally, exposure to SHS itself may induce psychological stress. Research indicates that SHS exposure is closely associated with heightened psychological stress, particularly among non-smokers^30^.

We observed that the association between exposure to SHS and depression symptoms was stronger in women than in men. Two studies conducted in South Korea on non-smoking adults demonstrated a positive association between SHS exposure and depression symptoms in women, but not in men^31^. Conversely, a nationwide population-based survey in Germany found that men exposed to SHS were less likely to be diagnosed with depression symptoms, while no significant association was observed in women^32^. These findings support the hypothesis that women may be more susceptible to SHS-related factors. Although the underlying biological mechanisms remain unclear, they may be linked to women’s hormonal cycles, particularly the fluctuations in estrogen and progesterone that are closely associated with emotional regulation^12^. Harmful chemicals in SHS may further exacerbate the effects of hormonal fluctuations on mood by influencing neurotransmitter systems and increasing their sensitivity to SHS exposure^13^. Another possible explanation is that the higher prevalence of smoking among men results in greater SHS exposure for women^33,34^. Additionally, various gender-related factors, such as childcare responsibilities and disparities in earning power, can impact women’s exposure to SHS and their ability to manage it^33^.

### Strengths and limitations

This study extends the investigation of the relationship between multiple indoor SHS environments (e.g. cars and other indoor areas) and symptoms of depression by distinguishing various sources of exposure in a more nuanced and accurate manner than previous studies, which primarily focused on homes and workplaces^10-14^. Utilizing the NHANES database and a multistage probability sampling design enhances the reliability and representativeness of the data. Additionally, our use of cumulative SHS assessments improves the accuracy of exposure categorization compared to earlier studies that relied on individual SHS exposures. Based on these findings, we recommend that public health departments expand the scope of smoke-free policies to encompass e-cigarettes and other tobacco products, as well as private spaces such as cars. However, several limitations exist in this study. First, the cross-sectional design restricts the ability to infer causality from the observed associations, with the possibility that individuals with depression might be more likely to live in environments with secondhand smoke exposure. Second, while the PHQ-9 is a widely recognized tool for measuring depression symptoms^20^, it is not a substitute for a comprehensive a clinical diagnosis, and the study may not have sufficiently accounted for potential confounders, such as the duration of depression symptoms, the use of antidepressant medications, and residential area. As noted by Purtle et al.^35^, urban residents may be more susceptible to developing depression compared to those in rural areas, which could have influenced our findings. Additionally, some subgroup analyses had limited sample sizes, which may reduce the precision of specific estimates. Third, our assessment of SHS exposure in the seven indoor environments was limited to a 7-day recall period, which may not adequately capture the effects of chronic exposure on depressive symptoms. Finally, the reliance on self-reported data, rather than biomarkers, to assess SHS exposure may compromise the objectivity, and our US-based findings may have limited applicability to countries with different cultural backgrounds, smoking regulations, and healthcare systems. Nevertheless, our sensitivity analyses adjusted for serum cotinine levels to address these limitations and mitigate potential bias in self-reported data^25,36^.

## CONCLUSIONS

Our study involving 6272 general US adults found that individuals exposed to SHS in cars and other indoor environments, as well as in areas where e-cigarettes are used, were more likely to report symptoms of depression compared to those not exposed to SHS. Notably, the prevalence of depression symptoms was significantly higher among individuals exposed to multiple sources of SHS. Further longitudinal research will be necessary to validate these findings.

## Supplementary Material



## Data Availability

The data supporting this research are available from the following source: https://www.cdc.gov/nchs/nhanes/index.htm.
