# Supplementary material for: Exploring the relationship between secondhand smoke exposure in different indoor environments and depression symptoms among non-smoking adults: A cross-sectional study from NHANES
Source: Tob Induc Dis. 2025 Aug 28;23:10.18332/tid/207154. doi: 10.18332/tid/207154 (PMC12396190; doi:10.18332/tid/207154)
Supplement: Supplementary file 1 [file TID-23-120-s1.pdf]

Supplementary Materials

Exploring the relationship between secondhand smoke exposure in different indoor environments and depression symptom among non-smoking adults: A cross-sectional study from NHANES

List of Content

Supplementary Figure S1 Pearson correlation matrix depicting the relationships among key covariates adjusted in the logistic regression analysis, NHANES 2013–2020 (n = 6,272)..**3**

Supplementary Table S1 Characteristics of the study population stratified by gender, NHANES 2013–2020 (n = 6,272). **4**

Supplementary Table S2 Characteristics of the study population stratified by race/ethnicity, NHANES 2013–2020 (n = 6,272)..**6**

Supplementary Table S3 Association between exposure to SHS and depression symptom in gender-stratified logistic regression models, NHANES 2013–2020 (n = 6,272). **8**

Supplementary Table S4 Association between exposure SHS and depression symptom in race/ethnicity-stratified logistic regression models, NHANES 2013–2020 (n = 6,272). **9**

Supplementary Table S5 Associations between exposure to SHS and depression symptom in sensitivity analysis using logistic regression models, NHANES 2013–2020 (n = 6,272)..**10**

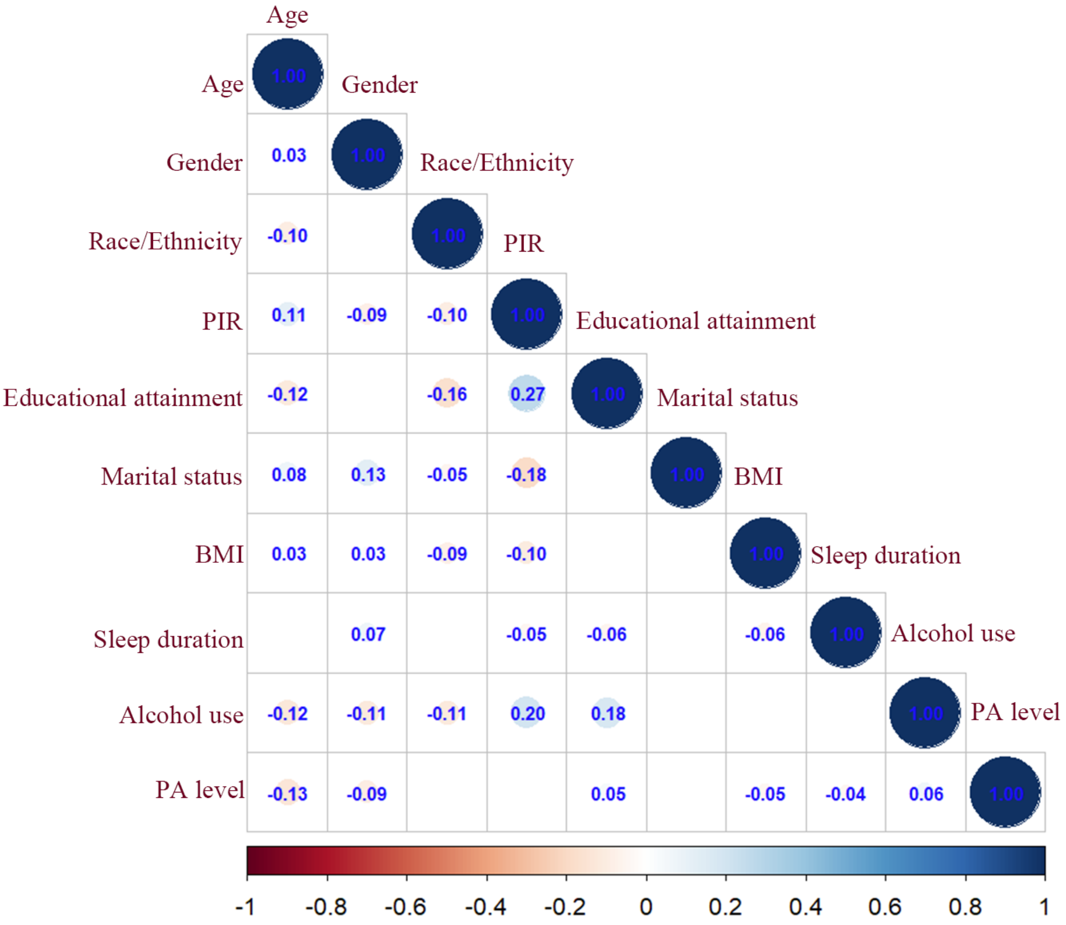

Supplementary Figure S1 Pearson correlation matrix depicting the relationships among key covariates adjusted in the logistic regression analysis, NHANES 2013–2020 (n = 6,272).

Abbreviations: BMI, Body mass index; NHANES, National Health and Nutrition Examination Survey; PA, Physical activity; PIR, Poverty income ratio.

Supplementary Table S1 Characteristics of the study population stratified by gender, NHANES 2013–2020 (n = 6,272).

Characteristic variables	Overall<br>Mean ± SE or N (%)	Male<br>Mean ± SE or N (%)	Female<br>Mean ± SE or N (%)	*P*-value[a]
**Total participants**	6,272 (100.0)	2,787 (46.1)	3,485 (53.9)	
**Age (years)**	44.24 ± 0.39	43.34 ± 0.42	45.01 ± 0.47	**< 0.001**
20-39	2,653 (43.8)	1,204 (44.4)	1,449 (43.2)	**< 0.001**
40-59	2,148 (35.8)	980 (38.4)	1,168 (33.5)	
≥ 60	1,471 (20.5)	603 (17.1)	868 (23.3)	
**Race/Ethnicity**				0.095
Non-Hispanic White	2,170 (64.2)	1,001 (64.9)	1,169 (63.6)	
Non-Hispanic Black	1,400 (11.0)	625 (10.4)	775 (11.4)	
Mexican American	900 (9.1)	372 (9.1)	528 (9.1)	
Other races (including multi-racial, other Hispanic)	1,802 (15.7)	789 (15.6)	1,013 (15.8)	
**Educational attainment**				0.107
Less than 9th grade	367 (2.8)	158 (2.8)	209 (2.8)	
9-11th grade (Includes 12th grade with no diploma)	456 (4.5)	202 (4.7)	254 (4.3)	
High school graduate/GED or equivalent	1,160 (18.6)	532 (20.0)	628 (17.3)	
Some college or AA degree	2,027 (31.0)	833 (29.4)	1,194 (3423)	
College graduate or above	2,262 (43.2)	1,062 (43.1)	1,200 (43.3)	
**Marital status**				**< 0.001**
Married/living with partner	3,888 (66.3)	1,860 (69.7)	2,028 (63.3)	
Never married	1,406 (21.0)	656 (22.3)	750 (19.8.5)	
Widowed/divorced/separated	978 (12.7)	271 (8.0)	707 (16.8)	
**PIR**	3.34 ± 0.05	3.46 ± 0.06	3.24 ± 0.06	**< 0.001**
Below poverty (< 1.0)	1,025 (10.6)	384 (9.0)	641 (12.0)	**< 0.001**
Above poverty (≥ 1.0)	5,247 (89.4)	2,403 (91.0)	2,844 (88.0)	
**BMI (kg/m^2)**	28.99 ± 0.16	29.07 ± 0.18	28.92 ± 0.22	0.527
< 25	1,876 (31.0)	749 (24.6)	1,127 (36.5)	**< 0.001**
≥ 25, < 30	1,963 (31.8)	1,035 (38.6)	928 (26.1)	
≥ 30	2,433 (37.2)	1,003 (36.8)	1,430 (37.5)	
**Sleep duration (hours)**	7.44 ± 0.03	7.31 ± 0.03	7.56 ± 0.04	**< 0.001**
< 7	1,730 (23.2)	844 (26.2)	886 (20.6)	**< 0.001**
≥ 7, < 9	3,554 (62.5)	1,582 (63.0)	1,972 (62.1)	
≥ 9	988 (14.3)	361 (10.8)	627 (17.2)	
**PA level**				**< 0.001**
Insufficient PA (< 600 MET)	1,099 (16.0)	376 (12.6)	723 (18.9)	
Sufficient PA (≥ 600 MET)	5,173 (84.0)	2,411 (87.4)	2,762 (81.1)	
**Alcohol use**				**< 0.001**
Never	1,181 (14.5)	375 (11.7)	806 (17.0)	
Former	438 (5.4)	212 (5.5)	226 (5.3)	
Current	4,653 (80.0)	2,200 (82.7)	2,453 (77.7)	
**Depression symptom**				**< 0.05**

No	5,921 (95.0)	2,669 (96.4)	3,252 (93.8)	
Yes	351 (5.0)	118 (3.6)	233 (6.2)	
**SHS in the workplace**				**< 0.001**
No	3,563 (91.9)	1,737 (89.2)	1,826 (94.9)	
Yes	405 (8.1)	279 (10.8)	126 (5.1)	
**SHS in restaurants**				0.219
No	3,905 (97.0)	1,772 (96.6)	2,133 (97.4)	
Yes	152 (3.0)	70 (3.4)	82 (2.6)	
**SHS in bars**				0.152
No	734 (84.3)	390 (82.5)	344 (86.4)	
Yes	203 (15.7)	110 (17.5)	93 (13.6.3)	
**SHS in cars**				0.203
No	5,216 (92.7)	2,313 (92.1)	2,903 (93.2)	
Yes	493 (7.3)	240 (7.9)	253 (6.8)	
**SHS in others' homes**				0.190
No	2,814 (90.7)	1,193 (89.8)	1,621 (91.4)	
Yes	384 (9.3)	190 (10.2)	194 (8.6)	
**SHS in other indoor areas**				**< 0.05**
No	3,793 (94.3)	1,654 (93.0)	2,139 (95.5)	
Yes	301 (5.7)	170 (7.0)	131 (4.5)	
**E-cigarette SHS indoors**				**< 0.001**
No	2,140 (86.3)	914 (83.2)	1,226 (88.8)	
Yes	340 (13.7)	185 (16.8)	155 (11.2)	
**Cumulative SHS exposure[b]**				**< 0.001**
Without SHS exposure	4,658 (78.4)	1,945 (74.2)	2,713 (82.0)	
Exposure to 1-2 SHS	1,274 (18.8)	667 (22.3)	607 (15.9)	
Exposure to ≥ 3 SHS	196 (2.7)	114 (3.5)	82 (2.1)	

[a] *P*-values are calculated using chi-square and t-tests; bold values indicate statistical significance.

[b] Cumulative SHS exposure refers to participants' simultaneous exposure to multiple SHS environments over the past 7 days. These environments include workplaces, restaurants, bars, cars, others' homes, other indoor areas, and exposure to e-cigarettes.

Abbreviations: AA, Associate's degree; BMI, Body mass index; GED, General equivalent diploma; MET, Metabolic equivalent; NHANES, National Health and Nutrition Examination Survey; PA, Physical activity; PIR, Poverty income ratio; SE, Standard error; SHS, Secondhand smoke.

Supplementary Table S2 Characteristics of the study population stratified by race/ethnicity, NHANES 2013–2020 (n = 6,272).

Characteristics variables	Overall<br>Mean ± SE or N (%)	Non-Hispanic White<br>Mean ± SE or N (%)	Non-Hispanic Black<br>Mean ± SE or N (%)	Mexican American<br>Mean ± SE or N (%)	Other races<br>Mean ± SE or N (%)	*P*-value[a]
**Total participants**	6,272 (100.0)	2,170 (64.2)	1,400 (11.0)	900 (9.1)	1,802 (15.7)	
**Age (years)**	44.24 ± 0.39	46.56 ± 0.50	40.49 ± 0.48	38.31 ± 0.50	40.80 ± 0.61	**< 0.001**
20-39	2,653 (43.8)	845 (38.3)	615 (51.8)	413 (58.4)	780 (52.0)	**< 0.001**
40-59	2,148 (35.8)	665 (36.6)	505 (35.3)	328 (33.9)	650 (33.8)	
≥ 60	1,471 (20.5)	660 (25.1)	280 (12.9)	159 (7.7)	372 (14.3)	
**Gender**						0.477
Male	2,787 (46.1)	1,001 (46.6)	625 (43.8)	372 (46.2)	789 (45.6)	
Female	3,485 (53.9)	1,169 (53.4)	775 (56.2)	528 (53.8)	1,013 (54.4)	
**Educational attainment**						**< 0.001**
Less than 9th grade	367 (2.8)	20 (0.5)	28 (1.7)	177 (13.6)	142 (6.5)	
9-11th grade (Includes 12th grade with no diploma)	456 (4.5)	76 (2.4)	95 (5.9)	160 (15.6)	125 (5.8)	
High school graduate/GED or equivalent	1,160 (18.6)	386 (17.4)	326 (24.1)	195 (25.3)	253 (15.6)	
Some college or AA degree	2,027 (31.0)	744 (30.5)	565 (39.1)	253 (30.4)	465 (27.4)	
College graduate or above	2,262 (43.2)	944 (49.2)	386 (29.1)	115 (15.2)	817 (44.7)	
**Marital status**						**< 0.001**
Married/living with partner	3,888 (66.3)	1,442 (70.0)	630 (45.4)	613 (67.1)	1,203 (65.0)	
Never married	1,406 (21.0)	375 (17.3)	482 (36.7)	164 (21.9)	385 (24.5)	
Widowed/divorced/separated	978 (12.7)	353 (12.7)	288 (17.9)	123 (11.0)	214 (10.5)	
**PIR**	3.34 ± 0.05	3.70 ± 0.06	2.63 ± 0.08	2.23 ± 0.09	3.02 ± 0.08	**< 0.001**
Below poverty (< 1.0)	1,025 (10.6)	206 (5.7)	266 (19.2)	251 (25.2)	302 (16.1)	**< 0.001**
Above poverty (≥ 1.0)	5,247 (89.4)	1,964 (94.3)	1,134 (80.8)	649 (74.8)	1,500 (83.9)	
**BMI, kg/m^2**	28.99 ± 0.16	28.69 ± 0.20	31.21 ± 0.30	30.71 ± 0.26	27.67 ± 0.21	**< 0.001**
< 25	1,876 (31.0)	697 (32.7)	295 (22.4)	155 (18.1)	729 (37.6)	**< 0.001**
≥ 25, < 30	1,963 (31.8)	686 (32.1)	394 (28.7)	297 (32.2)	586 (32.7)	
≥ 30	2,433 (37.2)	787 (35.2)	711 (48.9)	448 (49.7)	487 (29.7)	
**Sleep duration (hours)**	7.44 ± 0.03	7.52 ± 0.03	7.07 ± 0.06	7.41 ± 0.09	7.42 ± 0.04	**< 0.001**
< 7	1,730 (23.2)	441 (19.0)	567 (39.0)	252 (29.8)	470 (25.6)	**< 0.001**
≥ 7, < 9	3,554 (62.5)	1,396 (67.3)	616 (45.5)	496 (55.2)	1,046 (59.2)	
≥ 9	988 (14.3)	333 (13.7)	217 (15.5)	152 (15.1)	286 (15.3)	
**PA level**						0.358
Insufficient PA (< 600 MET)	1,099 (16.0)	371 (16.1)	230 (14.7)	157 (14.4)	341 (17.5)	
Sufficient PA (≥ 600 MET)	5,173 (84.0)	1,799 (83.9)	1,170 (85.3)	743 (85.6)	1,461 (82.5)	
**Alcohol use**						0.269
Never	1,181 (14.5)	297 (12.1)	256 (17.8)	183 (15.9)	445 (21.4)	
Former	438 (5.4)	150 (5.0)	90 (6.1)	71 (6.4)	127 (6.1)	
Current	4,653 (80.0)	1,723 (82.9)	1,054 (76.1)	646 (77.7)	1,230 (72.5)	
**Depression symptom**						0.642
No	5,921 (95.0)	2,054 (95.5)	1,320 (94.5)	842 (94.6)	1,705 (93.8)	
Yes	351 (5.0)	116 (4.5)	80 (5.5)	58 (5.4)	97 (6.2)	
**SHS in the workplace**						**< 0.001**
No	3,563 (91.9)	1,221 (94.4)	829 (87.7)	470 (82.4)	1,043 (90.5)	
Yes	405 (8.1)	83 (5.6)	126 (12.3)	99 (17.6)	97 (9.5)	
**SHS in restaurants**						**< 0.05**
No	3,905 (97.0)	1,518 (97.7)	768 (94.4)	555 (95.4)	1,064 (96.3)	
Yes	152 (3.0)	36 (2.3)	47 (5.6)	27 (4.6)	42 (3.7)	
**SHS in bars**						**< 0.001**
No	734 (84.3)	345 (88.4)	142 (66.0)	68 (68.9)	179 (82.0)	
Yes	203 (15.7)	54 (11.6)	78 (34.0)	27 (31.1)	44 (18.0)	
**SHS in cars**						**< 0.001**
No	5,216 (92.7)	1,862 (93.8)	1,104 (84.6)	740 (93.5)	1,510 (93.4)	
Yes	493 (7.3)	150 (6.2)	207 (15.4)	49 (6.5)	87 (6.6)	
**SHS in others' homes**						**< 0.001**
No	2,814 (90.7)	1,056 (92.6)	560 (78.7)	420 (89.8)	778 (91.4)	
Yes	384 (9.3)	109 (7.4)	161 (21.3)	50 (10.2)	64 (8.6)	
**SHS in other indoor areas**						**< 0.001**
No	3,793 (94.3)	1,544 (95.7)	751 (86.7)	426 (91.5)	1,072 (94.1)	

Yes	301 (5.7)	79 (4.3)	119 (13.3)	39 (8.5)	64 (5.9)	
**E-cigarette SHS indoors**						0.354
No	2,140 (86.3)	6,96 (87.1)	531 (84.4)	263 (85.4)	650 (87.4)	
Yes	340 (13.7)	103 (12.9)	98 (15.6)	45 (14.6)	94 (12.6)	
**Cumulative SHS Exposure[b]**						**< 0.001**
Without SHS exposure	4,658 (78.4)	1,714 (81.7)	894 (65.5)	639 (70.4)	1,411 (78.5)	
Exposure to 1-2 SHS	1,274 (18.8)	380 (16.2)	390 (28.1)	212 (26.9)	292 (18.7)	
Exposure to ≥ 3 SHS	196 (2.7)	43 (2.1)	93 (6.4)	20 (2.8)	40 (2.9)	

[a] *P*-values are calculated using chi-square and t-tests; bold values indicate statistical significance.

[b] Cumulative SHS exposure refers to participants' simultaneous exposure to multiple SHS environments over the past 7 days. These environments include workplaces, restaurants, bars, cars, others' homes, other indoor areas, and exposure to e-cigarettes.

Abbreviations: AA, Associate's degree; BMI, Body mass index; GED, General equivalent diploma; MET, Metabolic equivalent; NHANES, National Health and Nutrition Examination Survey; PA, Physical activity; PIR, Poverty income ratio; SE, Standard error; SHS, Secondhand smoke.

Supplementary Table S3 Association between exposure to SHS and depression symptom in gender-stratified logistic regression models, NHANES 2013–2020 (n = 6,272).

Exposure	Male							Female						
	Case/participants	Crude model[a] COR (95%CI)	*P*-value[b]	Model 1[a] AOR (95%CI)	*P*-value	Model 2[a] AOR (95%CI)	*P*-value[b]	Case/participants	Crude model[a] COR (95%CI)	*P*-value[b]	Model 1[a] AOR (95%CI)	*P*-value[b]	Model 2[a] AOR (95%CI)	*P*-value[b]
**SHS in the workplace**														
No	47/1,737	Reference		Reference		Reference		92/1,826	Reference		Reference		Reference	
Yes	11/279	1.48 (0.76, 2.88)	0.254	1.44 (0.73, 2.86)	0.291	1.04 (0.51, 2.11)	0.915	11/126	1.80 (0.94, 3.46)	0.077	1.81 (0.93, 3.50)	0.079	1.54 (0.77, 3.08)	0.222
**SHS in restaurants**														
No	64/1,772	Reference		Reference		Reference		114/2,133	Reference		Reference		Reference	
Yes	4/70	1.62 (0.57, 4.57)	0.365	1.58 (0.56, 4.49)	0.390	1.26 (0.42, 3.77)	0.681	8/82	1.91 (0.90, 4.07)	0.058	1.84 (0.86, 3.92)	0.117	1.73 (0.79, 3.77)	0.168
**SHS in bars**														
No	13/390	Reference		Reference		Reference		18/344	Reference		Reference		Reference	
Yes	7/110	1.97 (0.77, 5.07)	0.160	1.85 (0.70, 4.92)	0.215	1.50 (0.54, 4.19)	0.436	11/93	**2.43 (1.10, 5.34)**	**< 0.05**	2.23 (0.97, 5.14)	0.060	2.29 (0.93, 5.65)	0.071
**SHS in cars**														
No	88/2,313	Reference		Reference		Reference		176/2,903	Reference		Reference		Reference	
Yes	19/240	**2.17 (1.30, 3.64)**	**< 0.05**	**2.45 (1.42, 4.20)**	**< 0.05**	1.56 (0.88, 2.76)	0.129	34/253	**2.41 (1.63, 3.56)**	**< 0.001**	**2.38 (1.59, 3.58)**	**< 0.001**	**1.68 (1.10, 2.57)**	**< 0.05**
**SHS in others' homes**														
No	42/1,193	Reference		Reference		Reference		97/1,621	Reference		Reference		Reference	
Yes	13/190	**2.01 (1.06, 3.82)**	**< 0.05**	**2.21 (1.14, 4.28)**	**< 0.05**	1.29 (0.64, 2.59)	0.482	23/194	**2.11 (1.31, 3.42)**	**< 0.05**	**2.19 (1.34, 3.60)**	**< 0.05**	1.45 (0.85, 2.44)	0.169
**SHS in other indoor areas**														
No	53/1,654	Reference		Reference		Reference		125/2,139	Reference		Reference		Reference	
Yes	16/170	**3.14 (1.75, 5.62)**	**< 0.001**	**3.53 (1.93, 6.45)**	**< 0.001**	**2.65 (1.41, 4.98)**	**< 0.05**	15/131	**2.08 (1.18, 3.67)**	**< 0.05**	**2.05 (1.16, 3.64)**	**< 0.05**	1.59 (0.88, 2.87)	0.125
**E-cigarette SHS indoors**														
No	40/914	Reference		Reference		Reference		79/1226	Reference		Reference		Reference	
Yes	12/185	1.52 (0.78, 2.95)	0.221	1.51 (0.76, 3.00)	0.235	1.39 (0.68, 2.85)	0.368	20/155	**2.15 (1.28, 3.63)**	**< 0.05**	**1.93 (1.12, 3.33)**	**< 0.05**	**1.98 (1.12, 3.52)**	**< 0.05**
**Cumulative SHS Exposure[c]**														
Without SHS exposure	69/1,945	Reference		Reference		Reference		154/2,713	Reference		Reference		Reference	
Exposure to 1-2 SHS	39/667	**1.69 (1.13, 2.53)**	**< 0.05**	**1.81 (1.19, 2.74)**	**< 0.05**	1.33 (0.86, 2.06)	0.197	61/607	**1.86 (1.36, 2.53)**	**< 0.001**	**1.84 (1.34, 2.53)**	**< 0.001**	**1.51 (1.09, 2.10)**	**< 0.05**
Exposure to ≥ 3 SHS	7/114	1.78 (0.80, 3.96)	0.159	1.99 (0.88, 4.53)	0.100	1.37 (0.58, 3.24)	0.467	13/82	**3.13 (1.69, 5.79)**	**< 0.001**	**3.10 (1.65, 5.83)**	**< 0.001**	**2.33 (1.21, 4.50)**	**< 0.05**

[a] Crude model was unadjusted; Model 1 was controlling for age, race/ethnicity; Model 2 with additional adjustment for PIR, BMI, educational attainment, marital status, alcohol use, sleep duration, and PA level.

[b] Results of COR (95 %CI), AOR (95 %CI), *P*-value with bold valued were statistically significant.

[c] Cumulative SHS exposure refers to participants' simultaneous exposure to multiple SHS environments over the past 7 days. These environments include workplaces, restaurants, bars, cars, others' homes, other indoor areas, and exposure to e-cigarettes.

Abbreviation: AOR, Adjusted odd ratio; BMI, Body mass index; CI, Confidence interval; COR, Crude odds ratio; NHANES, National Health and Nutrition Examination Survey; PA, Physical activity; PIR, Poverty income ratio; SHS, Secondhand smoke.

Supplementary Table S4 Association between exposure SHS and depression symptom in race/ethnicity-stratified logistic regression models, NHANES 2013–2020 (n = 6,272).

Exposure	Non-Hispanic White							Non-Hispanic Black						
	Case/participants	Crude model[a] COR (95%CI)	*P*-value[b]	Model 1[a] AOR (95%CI)	*P*-value[b]	Model 2[a] AOR (95%CI)	*P*-value[b]	Case/participants	Crude model[a] COR (95%CI)	*P*-value[b]	Model 1[a] AOR (95%CI)	*P*-value[b]	Model 2[a] AOR (95%CI)	*P*-value[b]
**SHS in the workplace**														
No	48/1,221	Reference		Reference		Reference		30/829	Reference		Reference		Reference	
Yes	7/83	2.25 (0.99, 5.14)	0.05	**2.59 (1.12, 6.02)**	**< 0.05**	2.22 (0.91, 5.41)	**0.079**	5/126	1.10 (0.42, 2.89)	0.846	1.28 (0.48, 3.41)	0.626	1.18 (0.41, 3.38)	0.760
**SHS in restaurants**														
No	63/1,518	Reference		Reference		Reference		37/768	Reference		Reference		Reference	
Yes	1/36	0.66 (0.09, 4.89)	0.579	0.68 (0.09, 5.03)	0.703	0.41 (0.05, 3.28)	0.402	3/47	1.35 (0.40, 4.54)	0.631	1.27 (0.37, 4.31)	0.700	1.49 (0.42, 5.33)	0.542
**SHS in bars**														
No	15/345	Reference		Reference		Reference		7/142	Reference		Reference		Reference	
Yes	2/54	0.85 (0.19, 3.81)	0.828	0.85 (0.18, 3.89)	0.832	0.48 (0.08, 2.69)	0.400	7/78	1.90 (0.64, 5.63)	0.246	1.90 (0.63, 5.72)	0.251	1.84 (0.50, 6.76)	0.356
**SHS in cars**														
No	90/1,862	Reference		Reference		Reference		58/1,104	Reference		Reference		Reference	
Yes	19/150	**2.86 (1.69, 4.83)**	**< 0.001**	**2.78 (1.61, 4.79)**	**< 0.001**	1.47 (0.82, 2.65)	0.200	15/207	1.41 (0.78, 2.54)	0.253	1.42 (0.78, 2.58)	0.254	1.06 (0.56, 2.01)	0.847
**SHS in others' homes**														
No	42/1,056	Reference		Reference		Reference		27/560	Reference		Reference		Reference	
Yes	18/109	**4.78 (2.64, 8.64)**	**< 0.001**	**4.63 (2.55, 8.41)**	**< 0.001**	**2.43 (1.25, 4.72)**	**< 0.05**	8/161	1.03 (0.46, 2.32)	0.939	1.03 (0.45, 2.34)	0.941	0.76 (0.32, 1.81)	0.528
**SHS in other indoor areas**														
No	81/1,544	Reference		Reference		Reference		29/751	Reference		Reference		Reference	
Yes	9/79	**2.32 (1.12, 4.82)**	**< 0.05**	**2.35 (1.13, 4.90)**	**< 0.05**	1.72 (0.79, 3.72)	0.171	12/119	**2.79 (1.38, 5.64)**	**< 0.05**	**3.14 (1.51, 6.52)**	**< 0.001**	**2.85 (1.30, 6.24)**	**< 0.05**
**E-cigarette SHS indoors**														
No	31/696	Reference		Reference		Reference		33/531	Reference		Reference		Reference	
Yes	15/103	**3.66 (1.90, 7.04)**	**< 0.001**	**3.45 (1.74, 6.87)**	**< 0.001**	**3.44 (1.63, 7.25)**	**< 0.05**	9/98	1.53 (0.71, 3.30)	0.126	1.63 (0.73, 3.62)	0.065	1.82 (0.78, 4.25)	0.166
**Cumulative SHS Exposure[c]**														
Without SHS exposure	75/1,1714	Reference		Reference		Reference		45/894	Reference		Reference		Reference	
Exposure to 1-2 SHS	34/380	**2.15 (1.41, 3.27)**	**< 0.001**	**2.24 (1.45, 3.45)**	**< 0.001**	1.46 (0.92, 2.32)	0.112	26/390	1.35 (0.82, 2.22)	0.241	1.40 (0.84, 2.33)	0.192	1.14 (0.67, 1.95)	0.628
Exposure to ≥ 3 SHS	7/43	**4.25 (1.83, 9.86)**	**< 0.001**	**4.29 (1.82, 10.09)**	**< 0.001**	**3.08 (1.24, 7.69)**	**< 0.05**	7/93	1.54 (0.67, 3.51)	0.309	1.67 (0.71, 3.91)	0.238	1.54 (0.64, 3.74)	0.339

Exposure	Mexican American							Other races						
	Case/participants	Crude model[a] COR (95%CI)	*P*-value[b]	Model 1[a] AOR (95%CI)	*P*-value[b]	Model 2[a] AOR (95%CI)	*P*-value[b]	Case/participants	Crude model[a] COR (95%CI)	*P*-value[b]	Model 1[a] AOR (95%CI)	*P*-value[b]	Model 2[a] AOR (95%CI)	*P*-value[b]
**SHS in the workplace**														
No	21/470	Reference		Reference		Reference		40/1,043	Reference		Reference		Reference	
Yes	5/99	1.14 (0.42, 3.09)	0.801	1.21 (0.43, 3.38)	0.720	1.02 (0.33, 3.18)	0.971	5/97	1.36 (0.52, 3.54)	0.525	1.57 (0.60, 4.14)	0.357	1.06 (0.38, 2.97)	0.905
**SHS in restaurants**														
No	32/555	Reference		Reference		Reference		46/1,064	Reference		Reference		Reference	
Yes	3/27	2.04 (0.58, 7.15)	0.264	1.81 (0.51, 6.43)	0.358	1.84 (0.47, 7.26)	0.385	5/42	**2.99 (1.12, 7.96)**	**< 0.05**	**2.98 (1.12, 7.94)**	**< 0.05**	2.68 (0.93, 7.68)	0.067
**SHS in bars**														
No	4/68	Reference		Reference		Reference		5/179	Reference		Reference		Reference	
Yes	3/27	2.00 (0.42, 9.60)	0.387	2.43 (0.47, 12.56)	0.290	7.56 (0.42, 135.75)	0.170	6/44	**5.49 (1.59, 18.94)**	**< 0.05**	**5.80 (1.60, 21.08)**	**< 0.05**	**4.90 (1.08, 22.17)**	**< 0.05**
**SHS in cars**														
No	47/740	Reference		Reference		Reference		69/1,510	Reference		Reference		Reference	
Yes	6/49	2.06 (0.83, 5.08)	0.118	**2.77 (1.09, 7.03)**	**< 0.05**	2.23 (0.83, 5.98)	0.112	13/87	**3.67 (1.94, 6.94)**	**< 0.001**	**3.58 (1.86, 6.90)**	**< 0.001**	**2.53 (1.23, 5.21)**	**< 0.05**
**SHS in others' homes**														
No	26/420	Reference		Reference		Reference		44/778	Reference		Reference		Reference	
Yes	2/50	0.63 (0.15, 2.74)	0.540	0.69 (0.16, 3.03)	0.625	0.54 (0.12, 2.49)	0.431	8/64	**2.38 (1.07, 5.31)**	**< 0.05**	**2.55 (1.12, 5.81)**	**< 0.05**	1.55 (0.63, 3.79)	0.337
**SHS in other indoor areas**														
No	20/426	Reference		Reference		Reference		48/1,072	Reference		Reference		Reference	
Yes	4/39	2.32 (0.75, 7.17)	0.144	2.39 (0.77, 7.42)	0.133	1.66 (0.48, 5.77)	0.425	6/64	2.21 (0.91, 5.37)	0.081	2.40 (0.98, 5.87)	0.056	1.47 (0.56, 3.87)	0.437
**E-cigarette SHS indoors**														
No	17/263	Reference		Reference		Reference		38/650	Reference		Reference		Reference	
Yes	3/45	1.03 (0.29, 3.68)	0.959	1.42 (0.38, 5.38)	0.603	0.89 (0.20, 3.99)	0.879	5/94	0.90 (0.35, 2.36)	0.838	0.78 (0.29, 2.10)	0.629	0.74 (0.26, 2.13)	0.578
**Cumulative SHS Exposure[c]**														
Without SHS exposure	42/639	Reference		Reference		Reference		61/1,411	Reference		Reference		Reference	
Exposure to 1-2 SHS	13/212	0.93 (0.49, 1.77)	0.821	1.15 (0.60, 2.23)	0.672	1.12 (0.56, 2.22)	0.747	27/292	**2.25 (1.41, 3.61)**	**< 0.001**	**2.38 (1.47, 3.85)**	**< 0.001**	**1.89 (1.13, 3.17)**	**< 0.05**
Exposure to ≥ 3 SHS	2/20	1.58 (0.35, 7.04)	0.549	2.20 (0.48, 10.15)	0.313	1.29 (0.26, 6.41)	0.758	4/40	2.46 (0.85, 7.13)	0.098	2.67 (0.90, 7.89)	0.077	1.62 (0.52, 5.05)	0.405

a Crude model was unadjusted; Model 1 was controlling for age, gender; Model 2 with additional adjustment for PIR, BMI, educational attainment, marital status, alcohol use, sleep duration, and PA level.

b Results of COR (95 %CI), AOR (95 %CI), *P*-value with bold valued were statistically significant.

c Cumulative SHS exposure refers to participants' simultaneous exposure to multiple SHS environments over the past 7 days. These environments include workplaces, restaurants, bars, cars, others' homes, other indoor areas, and exposure to e-cigarettes.

Abbreviation: AOR, Adjusted odd ratio; BMI, Body mass index; CI, Confidence interval; COR, Crude odds ratio; NHANES, National Health and Nutrition Examination Survey; PA, Physical activity; PIR, Poverty income ratio; SHS, Secondhand smoke.

Supplementary Table S5 Associations between exposure to SHS and depression symptom in sensitivity analysis using logistic regression models, NHANES 2013–2020 (n = 6,272).

Exposure	Adjusted for diabetes mellitus		Adjusted for hypertension		Adjusted for cardiovascular diseases		Adjusted for sedentary behavior		Adjusted for total energy intake		Adjusted for serum cotinine		Multiple imputation	
	**AOR (95% CI) Multivariable-adjusted model[a]**	***P*-value[b]**	**AOR (95% CI) Multivariable adjusted model[a]**	***P*-value[b]**	**AOR (95% CI) Multivariable-adjusted model[a]**	***P*-value[b]**	**AOR (95% CI) Multivariable-adjusted model[a]**	***P*-value[b]**	**AOR (95% CI) Multivariable-adjusted model[a]**	***P*-value[b]**	**AOR (95% CI) Multivariable-adjusted model[a]**	***P*-value[b]**	***AOR (95% CI)* Multivariable-adjusted modela**	***P-value[b]***
**SHS in the workplace**														
No	Reference		Reference		Reference		Reference		Reference		Reference		Reference	
Yes	1.36 (0.83, 2.23)	0.228	1.28 (0.78, 2.10)	0.323	1.29 (0.79, 2.11)	0.313	1.29 (0.79, 2.12)	0.313	1.46 (0.88, 2.41)	0.139	1.22 (0.73, 2.05)	0.445	1.20 (0.81, 1.79)	0.365
**SHS in restaurants**														
No	Reference		Reference		Reference		Reference		Reference		Reference		Reference	
Yes	1.68 (0.89, 3.15)	0.110	1.55 (0.83, 2.93)	0.172	1.60 (0.85, 3.00)	0.146	1.60 (0.85, 3.01)	0.142	1.70 (0.90, 3.22)	0.100	1.45 (0.76, 2.80)	0.262	1.36 (0.84, 2.20)	0.212
**SHS in bars**														
No	Reference		Reference		Reference		Reference		Reference		Reference		Reference	
Yes	1.91 (0.99, 3.66)	0.052	1.85 (0.96, 3.54)	0.065	1.85 (0.96, 3.56)	0.064	1.86 (0.97, 3.59)	0.063	1.92 (0.99, 3.73)	0.056	1.85 (0.94, 3.62)	0.074	1.21 (0.70, 2.08)	0.493
**SHS in cars**														
No	Reference		Reference		Reference		Reference		Reference		Reference		Reference	
Yes	**1.60 (1.13, 2.26)**	**<0.05**	**1.65 (1.17, 2.32)**	**< 0.05**	**1.66 (1.18, 2.33)**	**< 0.05**	**1.64 (1.17, 2.30)**	**< 0.05**	**1.54 (1.07, 2.19)**	**< 0.05**	**1.58 (1.11, 2.25)**	**< 0.05**	**1.61 (1.24, 2.08)**	**< 0.05**
**SHS in others' homes**														
No	Reference		Reference		Reference		Reference		Reference		Reference		Reference	
Yes	1.41 (0.92, 2.15)	0.115	1.42 (0.93, 2.16)	0.103	1.42 (0.93, 2.16)	0.100	1.40 (0.92, 2.12)	0.120	1.38 (0.89, 2.14)	0.145	1.26 (0.81, 1.95)	0.306	1.27 (0.91, 1.79)	0.162
**SHS in other indoor areas**														
No	Reference		Reference		Reference		Reference		Reference		Reference		Reference	
Yes	**2.04 (1 33, 3.14)**	**< 0.05**	**2.02 (1.32, 3.09)**	**< 0.05**	**2.00 (1.31, 3.06)**	**< 0.05**	**2.04 (1.33, 3.11)**	**< 0.05**	**1.81 (1.16, 2.83)**	**< 0.05**	**2.00 (1.30, 3.08)**	**< 0.05**	**1.65 (1.18, 2.32)**	**< 0.05**
**E-cigarette SHS indoors**														
No	Reference		Reference		Reference		Reference		Reference		Reference		Reference	
Yes	**1.65 (1.06, 2.58)**	**< 0.05**	**1.75 (1.12, 2.72)**	**< 0.05**	**1.78 (1.14, 2.78)**	**< 0.05**	**1.77 (1.14, 2.75)**	**< 0.05**	**1.78 (1.13, 2.80)**	**< 0.05**	1.53 (0.95, 2.45)	0.080	**1.57 (1.10, 2.23)**	**< 0.05**
**Cumulative SHS Exposure[c]**														
Without SHS exposure	Reference		Reference		Reference		Reference		Reference		Reference		Reference	
Exposure to 1-2 SHS	**1.47 (1.13, 1.91)**	**< 0.05**	**1.46 (1.12, 1.89)**	**< 0.05**	**1.48 (1.14, 1.93)**	**< 0.05**	**1.46 (1.13, 1.90)**	**< 0.05**	**1.40 (1.07, 1.84)**	**< 0.05**	**1.39 (1.06, 1.82)**	**< 0.05**	**1.32 (1.22, 1.44)**	**< 0.001**
Exposure to ≥ 3 SHS	**1.98 (1.18, 3.33)**	**< 0.05**	**2.00 (1.19, 3.35)**	**< 0.05**	**1.97 (1.18, 3.30)**	**< 0.05**	**1.96 (1.17, 3.28)**	**< 0.05**	**1.99 (1.17, 3.37)**	**< 0.05**	**1.76 (1.02, 3.01)**	**< 0.05**	**1.69 (1.42, 2.01)**	**< 0.001**

[a] Multivariable-adjusted model was controlling for age, gender, race/ethnicity, educational attainment, BMI, PIR, marital status, alcohol use, sleep duration, and PA level.

[b] *P*-values and AOR in bold indicate statistical significance.

[c] Cumulative SHS exposure refers to participants' simultaneous exposure to multiple SHS environments over the past 7 days. These environments include workplaces, restaurants, bars, cars, others' homes, other indoor areas, and exposure to e-cigarettes.

© 2025 Liu Y. et al.
